# On the Nature and Treatment of Croup

**Published:** 1855-12

**Authors:** 


On the Nature and Treatment of Croup. By Dr. Honerkopf. The chief object of this paper is to call renewed attention to the efficacy of sulphate of copper in the treatment of croup. The little favor it has hitherto met with he believes due to its being considered a poisonous substance, and one, from its irritating nature, ill-suited to an inflammatory disease. As to the first point, he says he has employed it in 90 cases without any ill effects, although he administered 2846 grains, or an average of 31½ grains per case. In 15 cases the average reached 77 grains. One child took in 8 days 216 grains, or a daily average of 27 grains, and another 40 grains per diem for three days. No evil consequences resulted in any case, and convalescence was much more rapidly established than after anti-phlogistic treatment. In treating the poor, who are quite unable to pay for leeches, the economy of this plan is also worth consideration. In the author's cases the duration of the treatment varied from 1½ to 2 days, upon the average.

As to the other point, Dr. Honerkopf reviews the series of symptoms, in order to show that croup is not an inflammatory disease; but here we need not follow him, merely observing that when he states the effusion is pseudo-membrane is no essential feature of croup, and only occurs in about half the cases, that he must include in his 90 cases several that others would characterize as false or spasmodic croup, a far more curable affection. The great number of cures he met with (77 in 90 cases) also confirms this view. From some trials he has made the author is disposed to believe that small doses of the copper, given for a considerable period to children who are much predisposed to or who have already suffered from croup, may exert some prophylactic effect. As a curative agent he employs it solely, giving of a solution of from 6 to 8 grains in 1 oz. of distilled water, from a tea spoonful to a half or whole table-spoonful. The ease or difficulty with which vomiting is produced regulates the frequency with which the dose must be repeated; for the torpor of the nerves of the stomach increases with the progress of the disease, and the difficulty of producing vomiting becomes a measure of the severity of the affection. While at the beginning, in slight cases, and towards the decline of the disease, small doses of emetics suffice, much larger doses are required when the malady has continued some time, or is very severe; and after a certain point vomiting cannot be produced at all. A dose is given every 10 or 15 minutes, for four, six, or even eight times, till the more violent symptoms are abated, the quantity being regulated by the severity of the case. Occasionally such improvement is observed after the very first dose. The cough is first less troublesome, there are less anguish and dispnœa; and lastly, the croupy sound is exchanged for a mucous râle. The drug is now given in smaller doses every 20 or 30 minutes, and at still longer intervals. In bad cases, if even slight croupy sound remains, it is still continued every 2 hours, as a relapse is to be feared on the next night. It is also desirable that the patient should not be left without the means of meeting a recurrence, even on the second or third night; but a couple of doses will then usually suffice.

If in very bad cases we can scarcely perceive any change in the croupy sound in twelve or more hours, we must not yet despair, for the improvement is often sudden and unexpected. When the other symptoms have improved, but this tone remains, we must never relax, for the enemy only sleeps, and may break out again with renewed vigor. Everything depends upon the energy of the practitioner, who must at first see the doses forced down the child's throat, or they will be neglected, and especially as the child's condition and the friends' carelessness require watching the next evening or two, when relapse is so common. Although vomiting is not the object of the copper, and excited by other means has not like efficacy, it is the measure of its success. How far the remedy has to be carried depends upon the amount of success. A single grain exciting vomiting once or twice may suffice in slight cases, while in others 100 grains producing vomiting from 80 to 100 times may be necessary in others. Sometimes when the excitability of the stomach is quite exhausted, and large doses fail to excite vomiting, this may be roused again by giving 2 grs. of musk every few hours.

We have already said that cases of false croup must have been included among the author's 90; and we may here reproduce M. Guersant's summary of the various forms of angina. He observes that prior to his father's and M. Bretonneau's writings upon the subject, pseudo-membraneous angina, croup, and other affections were confounded together. Those writings and daily practice show that there are five distinct species of angina.

1. *False croup* (stridulous laryngitis) which comes on suddenly and terminates as a cold, not producing false membranes, and being almost always curable.

2. *Pharyngeal diphtheritis* is characterized by false membranes lining the tonsils and pharynx, to which they are usually confined. It is generally curable by caustic applications.

3. *True croup* (pseudo-membraneous largyngitis) is characterized by the development of false membranes in the larynx. These may be formed primarily in and confined to the larynx, or they may spread from the pharynx or trachea. This diphtheritic lesion, which is usually of slowish formation, and sometimes met with more than once in the same subject, is, so to say, localized, and produces death by asphyxia.

4. *General diphtheritis*, angina maligna. The true diphtheritis poison which kills the patient just as glanders kills the horse, is an insideous and contagious disease, and is indicated by great prostration, enlargement of the cervical glands, and deposition of false membranes in the nasal fossæ, the pharynx, sometimes the larynx and bronchi, the vulva, and on blistered surfaces. Neither this disease nor croup causes any gangrenous odor. It does not kill by asphyxia, so that tracheotomy is here contra-indicated. The disease is usually fatal, the patient sinking exhausted, and tonics with cauterization of the surfaces are the only means that offer any hopes of benefit.

5. The *true gangrenous angina* destroys any of the parts it attacks, as the tonsils, the uvula, the velum or pharynx, and may complicate any other angina just as all gangrenes may complicate or terminate all inflammations. This angina is far rarer than any other, is not accompanied at first by false membranes, and always ends fatally.

While on this subject we may notice that M. Marchal de Calvi recently read a paper to the Académie of Sciences, in which he recommends the administration of alkaline carbonates in what he terms *angine couenneuse*, meaning, we suppose, the pharyngeal diphtheritis of M. Guersant's arrangement. He regards it as a general disease localizing itself at certain parts of the mucous membranes, while cauterization acts merely locally, is difficult of performance, and sometimes injurious by reason of the irritation it induces. The most manifest phenomenon in this affection is the excess in the plasticity of the blood, and to this M. Marshal addresses his attention. He relates a case in which, besides leeching, 15 grains of bicarbonate of soda were given every half hour, the false membrane promptly disappearing.—*Lon. Lancet.*

